# Impact of HPV vaccine on CIN2+ recurrence after conization: a systematic review and meta-analysis of vaccination timing, valency and surgical margins

**DOI:** 10.3389/fonc.2026.1748972

**Published:** 2026-02-25

**Authors:** Mauro Francesco Pio Maiorano, Ambrogio Cazzolla, Brigida Anna Maiorano, Vera Loizzi, Gennaro Cormio, Giuseppe Colonna, Salvatore Lopez

**Affiliations:** 1 Unit of Obstetrics and Gynecology, Department of Interdisciplinary Medicine (DIM), University of Bari “Aldo Moro”, Polyclinic of Bari, Bari, Italy; 2 Unit of Oncologic Gynecology, IRCCS “Giovanni Paolo II” Oncologic Institute, Bari, Italy; 3 Department of Medical Oncology, IRCCS San Raffaele Hospital, Milan, Italy; 4 Translational Biomedicine and Neuroscience Department (DiBraiN), University of Bari “Aldo Moro”, Bari, Italy

**Keywords:** cervical cancer conization, Gardasil vaccine, HPV vaccine, HPV vaccine after conization, HPV vaccine CIN, HPV vaccine conization systematic review, human papillomavirus infection, cervical conization

## Abstract

**Background:**

Prophylactic Human Papillomavirus (HPV) vaccines prevent high-grade cervical lesions, but their role as adjuvant therapy after conization for high-grade cervical intraepithelial neoplasia (CIN2+) remains uncertain, particularly regarding vaccine type, timing, and margin status. This review aimed to quantify the effect of adjuvant HPV vaccination on CIN2+ recurrence after conization and to determine whether vaccine valency, timing of administration (before vs after surgery), and cone margin status modify this effect.

**Methods:**

We performed a PROSPERO-registered (CRD420251042109) systematic review and meta-analysis of randomized trials and cohort studies comparing HPV vaccination versus no vaccination in women treated with conization and/or loop electrosurgical excision procedure (LEEP) for histologically confirmed CIN2+. The primary outcome was recurrent CIN2+; secondary outcomes were HPV persistence/reinfection and vaccine-related adverse events. Random-effects models were used to pool risk ratios (RRs), with preplanned subgroup analyses by vaccine valency (bivalent, quadrivalent, nonavalent), timing of vaccination (before vs after conization), and cone margin status (positive vs negative), alongside sensitivity and GRADE certainty assessments.

**Results:**

Seventeen studies (four randomized controlled trials [RCTs], thirteen cohorts), including 33,181 women (6,665 vaccinated; 26,516 unvaccinated), met inclusion criteria. Overall, adjuvant HPV vaccination was associated with a 62% relative reduction in CIN2+ recurrence (pooled RR 0.38, 95% confidence interval [CI] 0.29-0.51; I² = 58.6%). Subgroup analyses showed similar benefit across valencies (bivalent RR 0.50, quadrivalent RR 0.37, nonavalent RR 0.41; p for subgroup difference = 0.94), vaccination before versus after conization (RR 0.57 vs 0.72; p = 0.35), and in both margin-negative (RR 0.34) and margin-positive (RR 0.40) women (p = 0.63). Data on HPV persistence suggested a predominantly prophylactic mechanism (prevention of new/re-infection rather than clearance), and no new safety signals emerged.

**Conclusions:**

Adjuvant prophylactic HPV vaccination meaningfully lowers CIN2+ recurrence after conization across vaccine types and clinical subgroups, supporting its integration into routine post-excisional care for eligible women as a low-burden strategy to reduce repeat procedures, preserve reproductive potential, and help avert progression to cervical cancer.

**Systematic Review Registration:**

https://www.crd.york.ac.uk/PROSPERO/view/CRD420251042109, identifier CRD420251042109.

## 1 Introduction

Cervical cancer remains a major global health concern, with persistent infection by high-risk human papillomavirus (HPV) types identified as the primary cause (1). Over the past two decades, the development and widespread implementation of prophylactic HPV vaccines, including bivalent, quadrivalent, and nonavalent formulations, have markedly reduced the incidence of high-grade cervical lesions and cervical cancer (2). Vaccination programs worldwide have demonstrated significant effectiveness, with recent national data showing reductions of over 80% in cervical cancer incidence among vaccinated cohorts (3–5). While the preventive benefits of HPV vaccination are well established, the potential role of vaccination in the secondary prevention of disease recurrence following surgical excision for high-grade cervical intraepithelial neoplasia (CIN2+) remains under investigation. Conization, although effective, does not eliminate the risk of recurrent or persistent disease, particularly among women with positive surgical margins or persistent HPV infection post-treatment (6–8). Emerging evidence suggests that HPV vaccination administered around the time of conization may enhance the clearance of residual HPV infection and reduce the risk of subsequent CIN2+ recurrence (9). Over the last decade, several quantitative syntheses have evaluated adjuvant HPV vaccination after local treatment for CIN. A 2021 meta-analysis of 11 studies reported that adding HPV vaccination to surgery was associated with roughly a 65% reduction in recurrent CIN2+ lesions (10). A subsequent systematic review of 22 studies similarly concluded that adjuvant vaccination significantly lowers recurrence, particularly for HPV16/18-related disease (11). More recently, the PAVIVE meta-analysis of 20 studies reported an overall vaccine effectiveness of about 70% against recurrent CIN2+ and suggested greater benefit when vaccination was initiated on or after the day of excision (12). However, these influential works did not incorporate the most recent nonavalent vaccine data and provided limited stratification by surgical margin status and timing within a unified analytical framework. Given these findings, and remaining uncertainties, it is critical to clarify whether HPV vaccination can serve as an adjuvant strategy following surgical treatment for CIN2+, and to define how its effectiveness might vary by vaccine valency, timing, and cone margin status. This systematic review and meta-analysis aimed to comprehensively evaluate the existing literature on the role of prophylactic HPV vaccination in reducing the risk of CIN2+ recurrence following conization, and to perform a meta-analysis to synthesize the available and most recent evidence. We aimed to evaluate the overall effectiveness of HPV vaccination and explore whether outcomes differ based on vaccine valency, vaccination timing relative to surgery, and margin status.

## 2 Methods

This systematic review and meta-analysis was conducted according to the recommendations of Cochrane Systematic Reviews, and our findings are reported following PRISMA and Meta-analysis of Observational Studies in Epidemiology (MOOSE) checklists (13, 14).

### 2.1 Eligibility criteria, information sources, and search strategy

A systematic review was conducted according to predefined eligibility criteria, based on the Population, Intervention, Comparison, and Outcome (PICO) framework (**Table 1**). We registered our systematic review on the international database of prospectively registered systematic reviews, PROSPERO (registration ID: CRD420251042109). Ethical approval was not required due to the inclusion of anonymized, previously published data. Studies were considered eligible if they included women diagnosed with histologically confirmed CIN2+, treated surgically by conization, loop electrosurgical excision procedure (LEEP), or cold knife conization, and compared outcomes between women receiving HPV vaccination (bivalent, quadrivalent, or nonavalent) versus unvaccinated controls. Vaccination could have occurred before or after conization. Studies lacking a control arm were excluded. The primary outcome of interest was histologically confirmed recurrence of CIN2+. Secondary outcomes included HPV persistence, HPV reinfection, and vaccine-related adverse events (AEs). Eligible study designs included randomized controlled trials (RCTs), as well as prospective and retrospective cohort studies published in peer-reviewed journals. Case reports, case series, reviews, commentaries, and conference abstracts without extractable clinical data were excluded. Only full-text articles published in English were considered. A comprehensive literature search was performed across PubMed, Embase, Scopus, and the Cochrane Library, covering studies published from 1 January 2013 up to 30 November 2025 without applying language restrictions at the database level. Non-English records identified by the search were not taken forward to full-text data extraction because we restricted inclusion to English-language publications. The search strategy included both Medical Subject Headings (MeSH) and free-text terms related to “HPV vaccine,” “cervical intraepithelial neoplasia,” “CIN2+,” “conization,” “recurrence,” and “vaccination after treatment.” Boolean operators (AND/OR) were used to combine search terms appropriately. Additionally, reference lists of relevant systematic reviews and included articles were screened manually to identify further eligible studies. The complete search strategies for PubMed, Embase, Scopus, and the Cochrane Library are provided in **Supplementary File 1** to ensure full reproducibility of the search.

**Table 1:** PICOS framework for studies’ eligibility.

Component	Descriptor
Population (P)	Women diagnosed with histologically confirmed cervical intraepithelial neoplasia grade 2 or worse (CIN2+), treated by conization, loop electrosurgical excision procedure (LEEP), or cold knife conization.
Intervention (I)	Administration of HPV vaccine (bivalent [2vHPV], quadrivalent [4vHPV], or nonavalent [9vHPV]) either before or after surgical treatment.
Comparison (C)	Unvaccinated control group.
Outcomes (O)	Primary Outcome: Histologically confirmed recurrence of CIN2 +. Secondary Outcomes: HPV persistence and/or reinfection, and vaccine-related adverse events (AEs).
Study Design (S)	Randomized controlled trials (RCTs), prospective and retrospective cohort studies

### 2.2 Study selection

**Figure 1:**
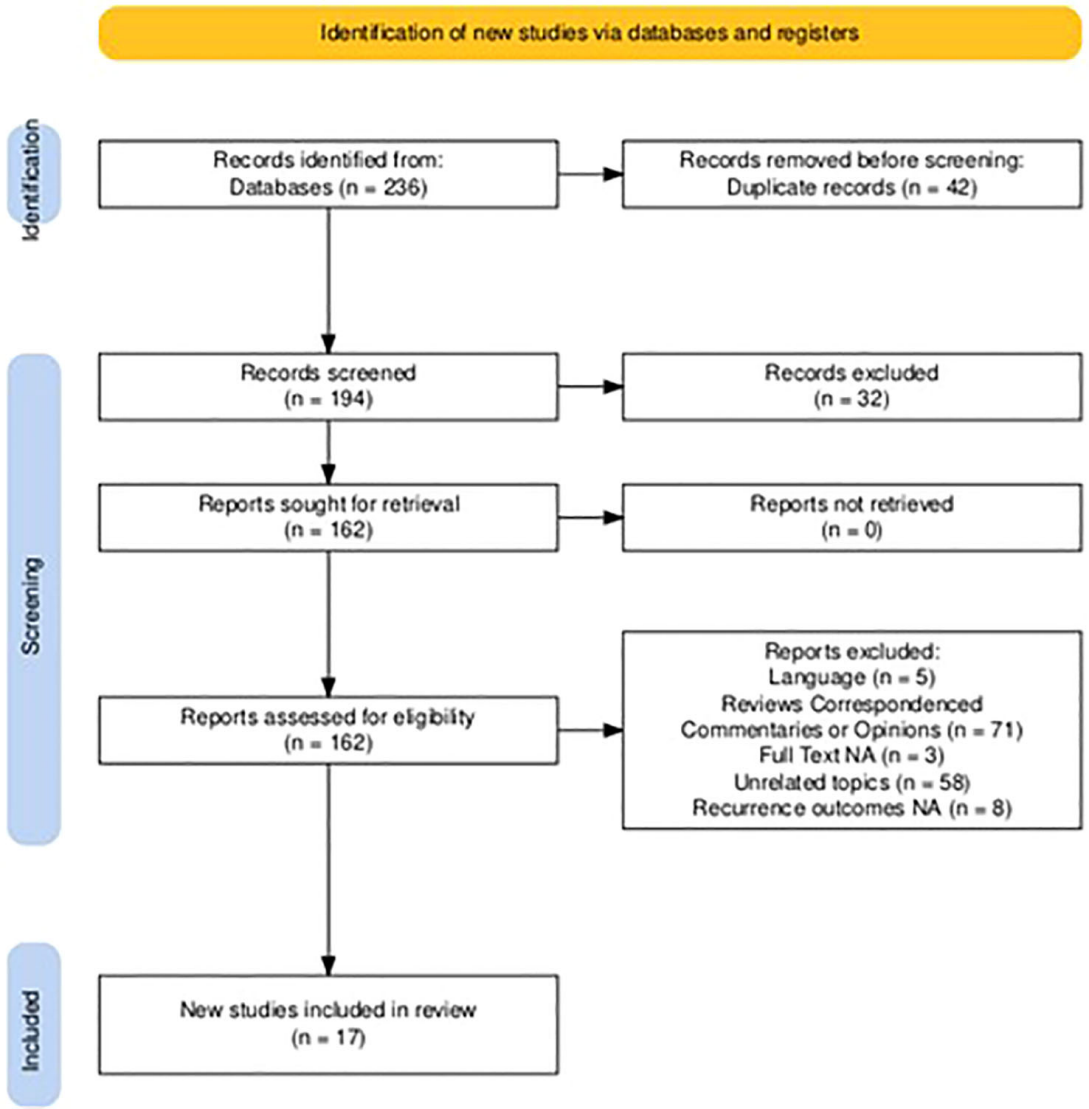
PRISMA flowchart for study selection. NA, not available.

Two reviewers (MFPM and AC) independently screened titles and abstracts retrieved from the initial search. Full-text articles were evaluated for eligibility based on the inclusion and exclusion criteria. Any disagreements were resolved by discussion and, if necessary, consultation with a third reviewer (SL). The study selection process followed the PRISMA guidelines, and a PRISMA flowchart was constructed to document the selection process (**Figure 1**) (13).

### 2.3 Data extraction

Data extraction was performed independently by two reviewers (MFPM and AC) using a standardized data collection form. Extracted information included: study characteristics (author, year, country, design), patient demographics (age, margin status), intervention details (type of HPV vaccine, timing of vaccination relative to conization), comparator group characteristics, follow-up duration, outcomes, including CIN2+ recurrence rates, HPV persistence, HPV reinfection, and adverse events. For each observational cohort, we noted whether follow-up for vaccinated women accrued from surgery, randomization, or vaccination, and we classified studies as being at higher risk of immortal-time bias when vaccination occurred after conization, but follow-up clearly started at surgery. When needed, corresponding authors were contacted for additional data.

### 2.4 Certainty of evidence (GRADE)

We assessed the certainty of evidence for each outcome using the GRADE approach, considering risk of bias, inconsistency, indirectness, imprecision, and publication bias. Evidence derived from randomized trials starts at high certainty; observational evidence starts at low, with rating down (−1/−2) for concerns and rating up (+1) for large effects (e.g., RR ≤ 0.5), dose-response, or where plausible residual confounding would reduce (not increase) the observed effect. Absolute effects were calculated using a representative baseline control risk of 94 per 1,000, derived from non-registry prospective control arms; we present relative effects (RR) alongside absolute risk differences and NNTs (inverse of absolute risk reduction).

### 2.5 Risk of bias assessment

Risk of bias was assessed independently by two reviewers (MFPM and SL) using the appropriate tools depending on study design. The Cochrane Risk of Bias 2.0 (RoB 2) tool was used for randomized controlled trials, while the Newcastle-Ottawa Scale (NOS) was applied for cohort studies (15, 16). Discrepancies were resolved through discussion and consensus. Risk of bias assessment considered selection bias, comparability of study groups, outcome assessment, and attrition bias.

### 2.6 Data synthesis

A meta-analysis was conducted using a random-effects model (DerSimonian and Laird method) to account for clinical and methodological heterogeneity across studies. Risk ratios (RRs) with 95% confidence intervals (CIs) were calculated for the primary outcome (17, 18). Subgroup meta-analyses were performed by: type of HPV vaccine (bivalent, quadrivalent, nonavalent), timing of vaccination relative to surgery (pre- vs. post-conization), and surgical margin status (positive vs. negative). Heterogeneity was assessed using Cochran’s Q test and quantified with the I² statistic, with I² values >50% indicating moderate to substantial heterogeneity (19). Sensitivity analyses were conducted by excluding individual studies and large registry-based datasets. Funnel plots were generated to assess publication bias. Statistical analyses were performed using SPSS version 24 and R version 4.4.2, employing the meta and metafor packages (20). A p-value of <0.05 was considered statistically significant. We also conducted exploratory random-effects meta-regression to investigate whether study-level characteristics explained between-study heterogeneity. Moderators included study design (randomized trial, observational cohort, registry-based), geographic region (Europe vs. non-Europe), timing of vaccination relative to conization (pre, post, mixed), and follow-up duration (months). Meta-regression models were conducted using the metareg function (20).

## 3 Results

A total of 236 studies were initially identified through the systematic search. After the removal of 42 duplicates, 194 studies remained for title and abstract screening. Following this initial screening, 162 studies were deemed potentially eligible for full-text evaluation. After applying the predefined eligibility criteria, 147 studies were excluded for the following reasons: 5 studies were written in languages other than English, 71 studies were identified as reviews, correspondences, commentaries, or expert opinions, 3 studies did not have a full-text version available, 58 studies focused on unrelated topics, including HPV vaccination in primary prevention settings or preclinical research, and 8 studies lacked extractable recurrence outcomes after conization. At the end of the selection process, 17 studies met the inclusion criteria and were included in the systematic review and meta-analysis (**Figure 1**) (21–37).

### 3.1 Characteristics of the included studies and overall main findings

This systematic review includes seventeen studies evaluating the role of HPV vaccination in preventing CIN2+ recurrence after conization (**Table 2**). The studies, conducted across diverse geographical regions, involved 33’181 women,of whom 6665 received HPV vaccination and 26’516 served as unvaccinated controls. Study designs varied: four studies were RCTs (22, 24, 27, 37), eight were prospective (21, 23, 25, 28, 31, 34), and five were retrospective cohort studies (26, 29, 30, 35, 36). The participants’ ages ranged from 28 to 39 years, and median follow-up durations extended from 2 to 5 years, with single cases followed up to 15 years. Regarding the vaccine types used, eight studies administered the quadrivalent vaccine (Gardasil^®^) (21, 23–25, 27, 29, 30, 34), three used the nonavalent vaccine (Gardasil 9^®^) (33, 35, 37), one reported outcomes from a subgroup of the bivalent vaccine (Cervarix^®^) (22), and five studies reported results from multiple or unknown types of vaccines (26, 28, 31, 32, 36). All studies assessed CIN2+ recurrence as a primary or secondary endpoint. The vaccine administration after the procedure occurred within 12 months after conization, though five studies started their administration before the procedure (25, 26, 28, 33, 36). Across studies, outcome definitions were broadly consistent in focusing on high-grade disease, but differed in whether they restricted events to strictly histologically confirmed recurrent CIN2+ or used broader composite endpoints that also captured persistent High-Grade Squamous Intraepithelial Lesion (HSIL) or Low-Grade Squamous Intraepithelial Lesion (LSIL)/HPV. Most cohorts (21, 23, 29–32, 34–37) defined the primary endpoint as histologic CIN2+ recurrence after the first post-treatment visit, whereas a minority (22, 26–28, 33) employed composite ‘persistent/recurrent HSIL/HPV’ outcomes or earlier risk windows.

**Table 2:** Summary of the studies’ results.

Study, year	Design	Valency, timing (B/A)	Sample (V vs C)	Follow-up (median)	CIN2+ recurrence	Key findings	Outcome definition
Kang et al., 2013​ (21)	Prospective	Quadrivalent (A)	360 vs 377	2 years	9/360 vs 27/377 (p < 0.01)	Vaccination halved recurrence; no-vaccine was an independent risk factor (HR = 2.840)	Histologically confirmed recurrent CIN2–3 after LEEP
Hildesheim et al., 2016 (22)	RCT	Bivalent (A)	362 vs 375	56.7 vs 27.3 months	3/362 vs 2/375 (p = 0.68)	No significant effect of vaccination on rates of infection/lesions after treatment	Post-treatment cervical lesions, with analyses focused on histologic CIN2+ after excisional treatment
Ghelardi et al., 2018 (23)	Prospective (case-control)	Quadrivalent (A)	172 vs 172	3 years	2/172 vs 11/172 (p < 0.01)	80% reduction in recurrences with vaccine (1.2% vs 6.4%)	Histologic CIN2+ after excisional treatment
Pieralli et al., 2018 (24)	RCT	Quadrivalent (A)	89 vs 89	3 years	0/89 vs 4/89	Significant reduction of overall HPV-related disease recurrence	Any histologically confirmed HPV-related disease recurrence (cervical, vaginal or vulvar)
Sand et al., 2020 (25)	Prospective	Quadrivalent (A 1675, B 399)	2,074 vs 15,054	4.5 years	82/2074 vs 777/15054 B vs A conization 14/399 vs 68/1675	No statistically significant difference (5-year CIN2+ risk 10% vs 12%)	Subsequent histologic CIN2+
Ortega-Quiñonero et al., 2019 (26)	Retrospective	Bi- (70) or Quadrivalent (33) (A 57, B 46)	103 vs 139	2 years	5/103 vs 22/139 (p = 0.01) B vs A conization 3/46 vs 2/57 Bi- vs quadrivalent 2/70 vs 3/33	Benefit was strongest for HPV16/18-related disease and with negative margins; no difference by vaccine type or timing.	Histologic recurrence of HSIL/CIN2-3 (or worse) after conization
Karimi-Zarchi et al., 2020​ (27)	RCT	Quadrivalent (A)	138 vs 104	2 years	24/138 vs 41/104 (p = 0.018)​	Significantly fewer persistent CIN in vaccinated arm: regression in 75.6% vs 45.7% of CIN1; overall 58.7% recurrence reduction. No AEs.	Persistent CIN1–3 at 24 months
del Pino et al., 2020​ (28)	Prospective	Bi- (30), Quadri- (7), Nonavalent (98). Unknown (18) (A 143, B 10)	153 vs 112	22.4 months	5/153 vs 12/112 (p = 0.015)	4.5-fold risk reduction (OR 0.20, 95% CI 0.1-0.7) with vaccination​; free vaccine policy improved uptake.	Persistent CIN1–3 at 24 months defined by histology or cytology
Bogani et al., 2020 (29)	Retrospective	Quadrivalent (A)	100 vs 200	5 years	2/100 vs 11/200	Trend toward lower 5-year recurrence with vaccine (2% vs 5.5%)	Histologic recurrent (not persistent) CIN2
Petrillo et al., 2020 (30)	Retrospective	Quadrivalent (A)	182 vs 103	6 months	6/182 vs 16/103 (p < 0.001)	Vaccination was independently protective (OR 0.4; 95% CI 0.2-0.8; p=0.02); effect stronger for severe lesions (OR 0.2; 95% CI 0.1-0.6; p=0.02).	Recurrent cervical dysplasia after LEEP
Gomez de la Rosa et al., 2021 (31)	Prospective	Bi- or Quadrivalent (A)	160 vs 171	6–48 months	4/160 vs 16/171 (p = 0.009)	74% of recurrence risk reduction with vaccination	Recurrent cervical dysplasia after LEEP
Casajuana-Pérez et al., 2022 (32)	Prospective	Bi- or Quadrivalent (A)	277 vs 286	29.6 vs 36.5 months	12/277 vs 28/286 (p = 0.014)	57% reduction of persistence/recurrence after treatment	Persistence/recurrence of HSIL/CIN2–3 after treatment, defined by histology at colposcopy-directed biopsy
Henere et al., 2022 (33)	Prospective	Nonavalent (A 193, B 113)	306 vs 92	9–31 months	6/306 vs 6/92 (p = 0.037) B vs A conization 1/113 vs 4/193 (p = 0.04)	Women vaccinated before treatment showed a lower rate of post-treatment HSIL (0.9% vs. 6.5%)	Post-treatment HSIL recurrence (CIN2+) confirmed histologically
Chen et al., 2023 (34)	Prospective	Quadrivalent (A)	148 vs 273	2 years	2/148 vs 29/273 (p < 0.01)	80% relative risk reduction (2% vs 10.6%); lower HPV re-infection in the V group.	Post-treatment HSIL recurrence (CIN2+) confirmed histologically
Dvořák et al., 2024 (35)	Retrospective	Nonavalent (A)	49 vs 98	30 ± 22 months	1/49 vs 16/98 (p = 0.038)	90% reduction in recurrence	Recurrent CIN2+ (HSIL) after LEEP
Petráˇs et al., 2025 (36)	Retrospective	Bi-, Quadri- or Nonavalent (A 919, B 502)	1421 vs 8464	4.2 ± 3.4 years	A 15/919 vs 513/8464 B 14/502 vs 513/8464	Vaccination significantly reduced recurrence, stronger effect post-excision	Histologically confirmed recurrent high-grade CIN2+ after excision,
van de Laar et al., 2025 (37)	RCT	Nonavalent (A)	402 vs 407	24 months	23/402 vs 35/407 (p = 0.11)	Lower recurrence with vaccination (5.7% vs 8.6%), not statistically significant	Pathologically confirmed recurrent CIN2–3 after LEEP, diagnosed via protocol-driven colposcopy/biopsy

A, after conization; adj. HR, adjusted Hazard Ratio; B, before conization; C, control; CI, Confidence Interval; CIN, Cervical Intraepithelial Neoplasia; CIN2+, Cervical Intraepithelial Neoplasia grade 2 or worse; HPV, Human Papillomavirus; HR, Hazard Ratio; IRR, Incidence Rate Ratio; n.s., not significant; OR, Odds Ratio; RCT, Randomized Controlled Trial; V, vaccinated.

### 3.2 CIN2+ recurrence outcomes

Across the 17 included studies, 12 reported a statistically significant reduction in CIN2+ recurrence with post-treatment HPV vaccination (21, 23, 26–28, 30–36). In Kang et al., recurrent CIN2–3 occurred in 2.5% (9/360) of vaccinated vs 7.2% (27/377) controls (p<0.01); lack of vaccination independently increased risk (21). In the prospective SPERANZA study, Ghelardi et al. reported a 4-year HSIL/CIN2+ recurrence of 1.2% in vaccinated women versus 6.4% in unvaccinated (p=0.011), corresponding to 81% clinical effectiveness (23). Del Pino et al. found 3.3% versus 10.7% persistent/recurrent HSIL (p=0.015); vaccination remained protective in multivariable analysis (adjusted odds ratio [AOR] 0.20, 95% confidence interval [CI] 0.1-0.7), and among women disease-free at first post-conization control, none recurred in the vaccinated group versus 6.1% of controls (p=0.032) (28). In the VENUS study cohort, Casajuana-Pérez et al. observed 4.3% vs 9.8% HSIL/CIN2–3 with vaccination (Hazard Ratio [HR] 0.43, 95% CI 0.22-0.84; p=0.014), with similarly low recurrence when the 6-month co-test was negative (1.1% vs 1.5%) (32). Bogani et al. (5-year, multicenter) reported a non-significant trend toward lower recurrence overall (1.7% vs 5.7%; p=0.068), but a significant reduction for recurrent (not persistent) lesions (0% vs 4.5%; p=0.031) (29). Chen et al., after a median follow-up of 30 months, recorded 2.03% vs 10.62% CIN2+ at 2 years; lack of vaccination independently increased recurrence risk (OR 12.35, 95% CI 1.92-79.49; p=0.008) (34). Sand et al. estimated a non-significant adjusted hazard ratio of 0.86 (95% CI 0.67-1.09) comparing vaccinated to unvaccinated women post-conization (25). Dvořák et al., assessing the nonavalent vaccine, reported an incidence rate ratio (IRR) of 0.10 (95% CI 0.012-0.99), indicating 90% lower CIN2+ recurrence; among women with positive margins, the reduction was also 42% but not significant (35). In a much larger population-based cohort from the same country, Petráš et al. followed 10,054 women for up to 15 years and found that post-excision vaccination reduced CIN2+ recurrence by 74% (adjusted IRR 0.26, 95% CI 0.15-0.43), and prophylactic vaccination by 54% (adjusted IRR 0.46, 95% CI 0.27-0.78); among women with positive margins, recurrence rates fell from 51.6 to 9.8 per 1000 person-years with post-excision vaccination (79% reduction) and to 14.9 per 1000 person-years with prophylactic vaccination (62% reduction) (36). Ortega-Quiñonero et al. found 4.8% recurrence among vaccinated patients versus 15.8% in unvaccinated; and protection was strongest in HPV16/18-related disease (p<0.05) (26). Gómez de la Rosa et al., after 48-month of follow-up, reported 2.5% vs 9.4% recurrence (p=0.009), with non-vaccination independently increasing risk 3.5-fold on multivariable Cox analysis (31). Henere et al. observed a significant lower HSIL recurrence when vaccination occurred before surgery (0.9% vs 6.5%), and among women with post-treatment high-risk (hr)HPV positivity (2.6% vs 10.5%) (33). The randomized trial by Karimi-Zarchi et al. reported a 58.7% efficacy of quadrivalent vaccination over 24 months (p=0.018), with women showing a greater reduction from CIN2–3 at follow-up in the vaccine arm (27). Petrillo et al. found any-grade recurrence in 7.1% vaccinated vs 16.5% unvaccinated (p=0.010), and specifically CIN2+/Carcinoma *in-situ* (CIS) in 3.3% vs 13.6% (p=0.001); vaccination remained protective (OR 0.40 for any recurrence; OR 0.20 for severe lesions) (30). In the randomized study by Pieralli et al., overall recurrences were 3.4% vaccinated vs 13.5% unvaccinated, and notably no high-grade cervical recurrences occurred among vaccinated women versus 4.5% in controls (24). By contrast, Hildesheim et al. observed no significant reduction in post-treatment infections or lesions; vaccine-efficacy estimates against CIN2+ after treatment were not significant and centered near null or negative (22). Similarly, the phase 4 VACCIN randomized placebo-controlled trial by van de Laar et al. did not demonstrate superiority of nonavalent vaccination after LEEP: CIN2–3 recurred in 6% of vaccinated vs 9% of placebo recipients at 24 months (RR 0.67, 95% CI 0.40-1.11; p=0.11) despite a non-significant trend toward fewer recurrences and additional LEEPs in the vaccine arm (37).

### 3.3 HPV persistence and reinfection

Of the 17 studies, 8 explicitly evaluated HPV persistence/reinfection after conization (22, 23, 28, 29, 32–34, 37). The outcome’s definitions varied: any hrHPV DNA positivity at fixed timepoints as a proxy for persistence/reinfection; genotype-specific persistence, and composite outcomes such as “persistent/recurrent LSIL/HPV.” Across these, one study showed a statistically significant reduction in post-treatment hrHPV positivity with vaccination (34), while several reported no difference in persistence despite clinical benefits (23, 32, 33), and one large randomized study suggested prophylaxis against incident infections but no effect on persistent infection after treatment (22). Particularly, Chen et al. reported significantly lower hrHPV positivity among vaccinated women at both 1 year (16.9% vs 25.3%, p = 0.049) and 2 years (8.1% vs 15.8%, p = 0.026) after LEEP, indicating reduced persistence/reinfection with vaccination (34). Ghelardi et al. (SPERANZA) emphasized that vaccination does not clear prevalent infection, as HPV tests at 6 months post-surgery showed no between-group differences, supporting a mechanism of preventing new infection/reactivation rather than therapeutic clearance (23). Del Pino et al. documented that 19.3% of the cohort had persistent/recurrent LSIL/HPV during follow-up, with vaccination linked to fewer subsequent HSIL events in key subgroups (28). On the other hand, Casajuana-Pérez et al. found no difference in persistent/recurrent HPV16/18 infection at end of follow-up (9.7% in both groups; p = 0.814), despite a vaccine-associated reduction in HSIL persistence/recurrence (32). Henere et al. reported similar HPV16/18 prevalence at end of follow-up among women vaccinated before/after conization, and unvaccinated (8.8% vs 5.7% vs 6.5%; p = 0.795), although post-treatment HSIL was less frequent with vaccination in patients with early HPV-positive controls (33). Bogani et al. recorded post-conization HPV persistence in a tested subset (17.5%); persistence was more often documented among vaccinated than unvaccinated women (44.0% vs 15.8%) (29). Finally Hildesheim et al. evaluated women post-LEEP and found no significant vaccine effect (VE) on persistent HPV16/18 infection after treatment (VE = 34.7%), but signals of protection against incident, new post-treatment infections (VE = 57.9% for incident HPV16/18; VE = 72.9% for incident HPV31/33/45; VE = 36.7% for incident oncogenic HPV), consistent with a reinfection-prevention effect rather than clearance of established infection (22). Consistently, the recent VACCIN randomized trial found no significant reduction in hrHPV positivity at 6 or 24 months after LEEP in vaccinated versus placebo-treated women, despite a modest, non-significant trend toward faster HPV clearance (25% vs 29% at 6 months [RR 0.84, p=0.12] and 32% vs 36% at 24 months [RR 0.87, p=0.15]) (37).

### 3.4 Safety outcomes

Safety outcomes reporting was sparse and largely descriptive. The RCT by Karimi-Zarchi et al. reported typical reactogenicity (headache in 9.7% of women receiving three doses; injection-site redness/rash in 90-97%), no additional complications, and no vaccine-related serious AEs; notably, a small number of participants were lost to follow-up citing, without specifying, “allergies to the vaccine” (27). The VACCIN RCT systematically collected solicited adverse events by structured telephone interviews after each dose, confirming the expected reactogenicity profile of the nonavalent vaccine: injection-site pain and redness were more frequent in the vaccine arm, whereas systemic symptoms were similar between groups, and no vaccine-related serious adverse events were observed (37). Hildesheim et al. likewise recorded local and systemic reactions consistent with the established safety profile of the bivalent vaccine and found no excess serious AEs in vaccinated women after treatment (22). Pieralli et al. explicitly reported no AEs or reactions in the vaccination group, supporting good tolerability in the post-treatment setting (24). No new or unexpected safety signals emerged in women vaccinated around the time of conization, and the majority of reported events were mild and local, consistent with known reactogenicity. However, AE assessment was not standardized, most series were underpowered to detect rare events, and many did not report safety outcomes at all, so the absence of new signals in this patients’ setting should be interpreted cautiously.

### 3.5 Meta-analyses

**Figure 2:**
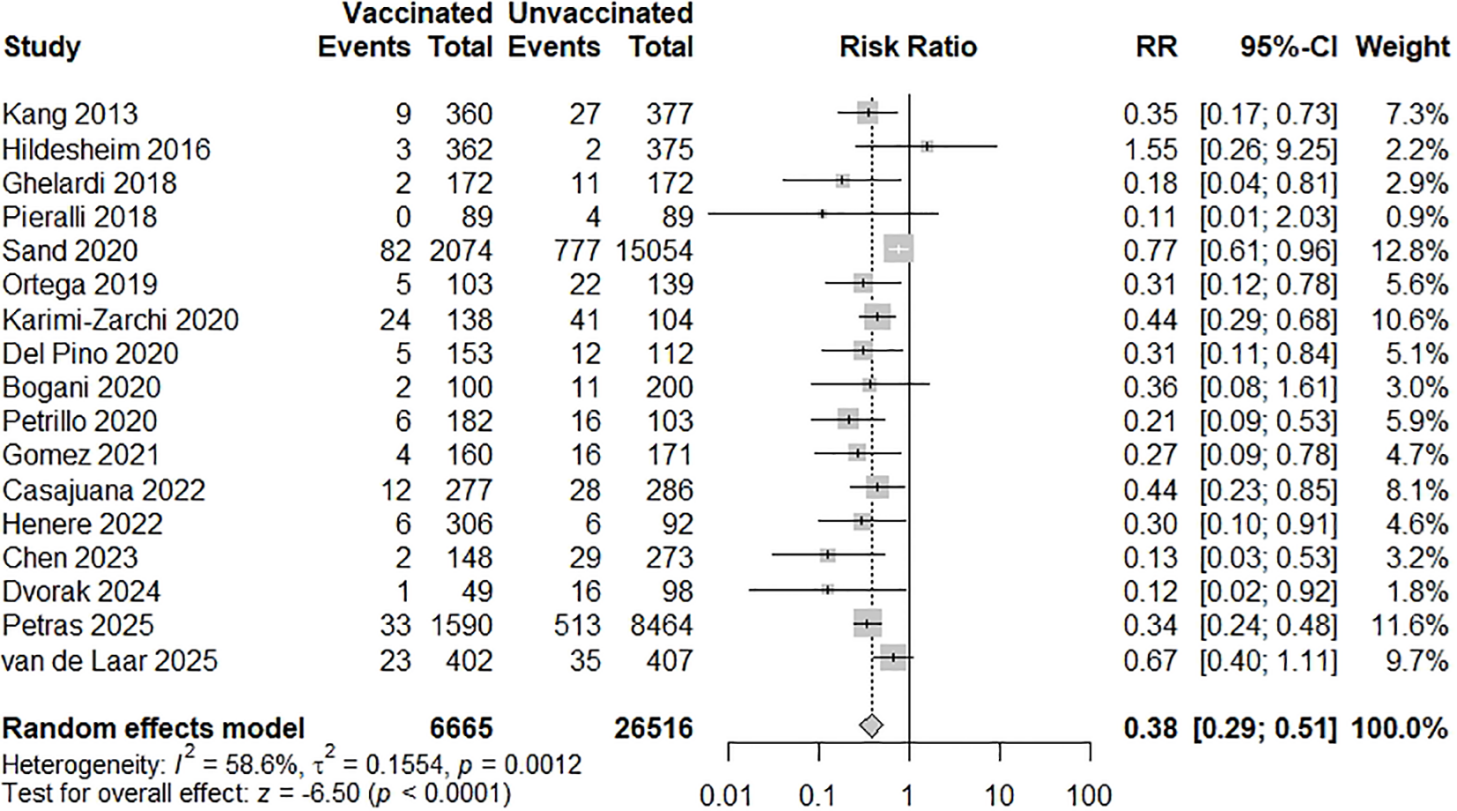
Forest plot of risk ratios for CIN2+ recurrence in vaccinated versus unvaccinated women after conization. HPV vaccination in the post-treatment setting significantly reduced recurrence risk (pooled RR = 0.38; 95% CI: 0.29-0.51; p < 0.001). Moderate heterogeneity was observed across studies (21–37).

**Figure 3:**
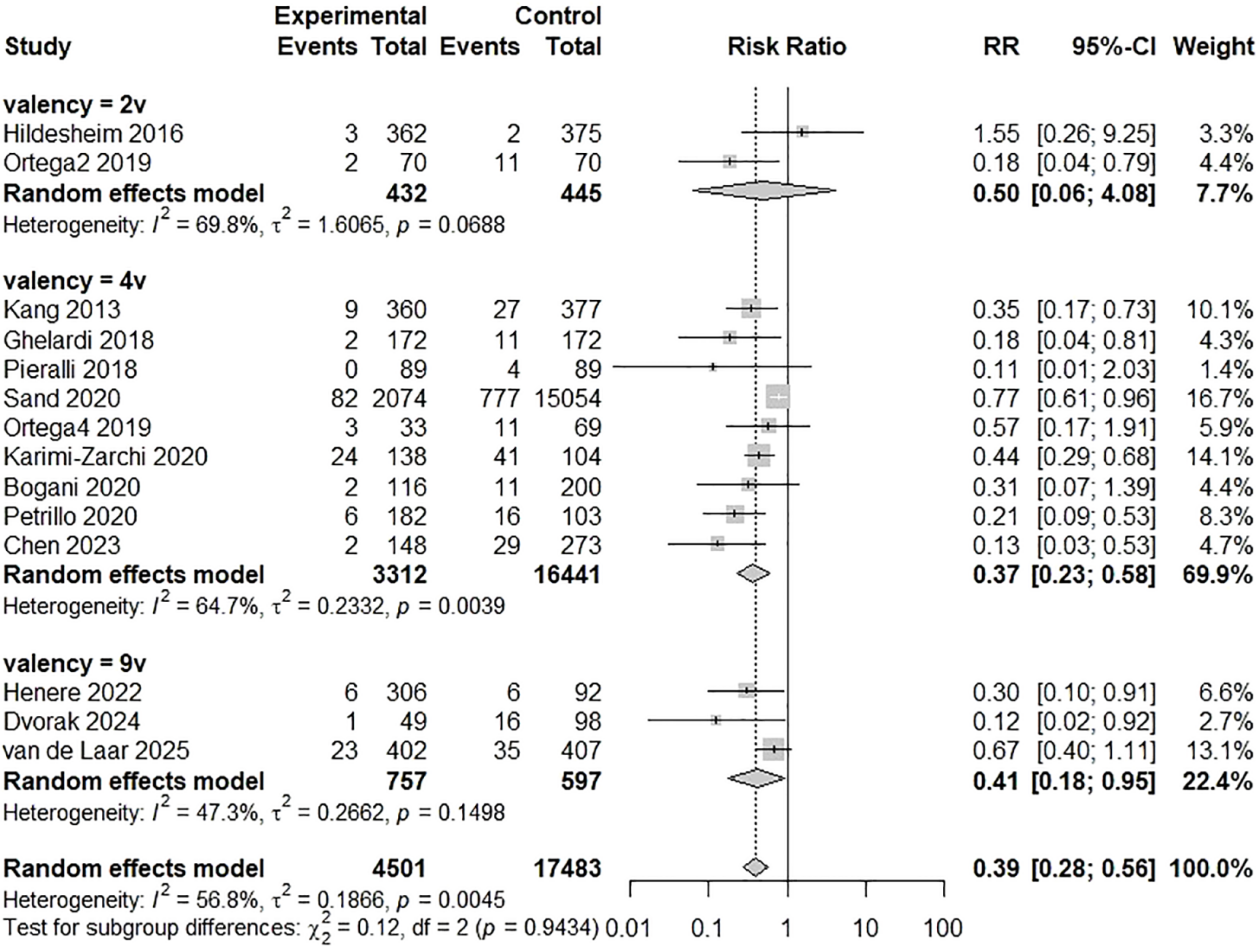
Subgroup meta-analysis of CIN2+ recurrence according to HPV vaccine valency. Random-effects pooled estimates: bivalent RR = 0.50 (95% CI 0.06-4.08; 2 cohorts), quadrivalent RR = 0.37 (95% CI 0.23-0.58; 9 studies), and nonavalent RR = 0.41 (95% CI 0.18-0.95; 3 studies). Overall pooled RR = 0.39 (95% CI 0.28-0.56); heterogeneity I² = 56.8%, τ² = 0.1866 (p = 0.0045). No evidence of subgroup differences by valency (χ² = 0.12, df = 2, p = 0.94) (21–27, 29, 30, 33–35, 37).

**Figure 4:**
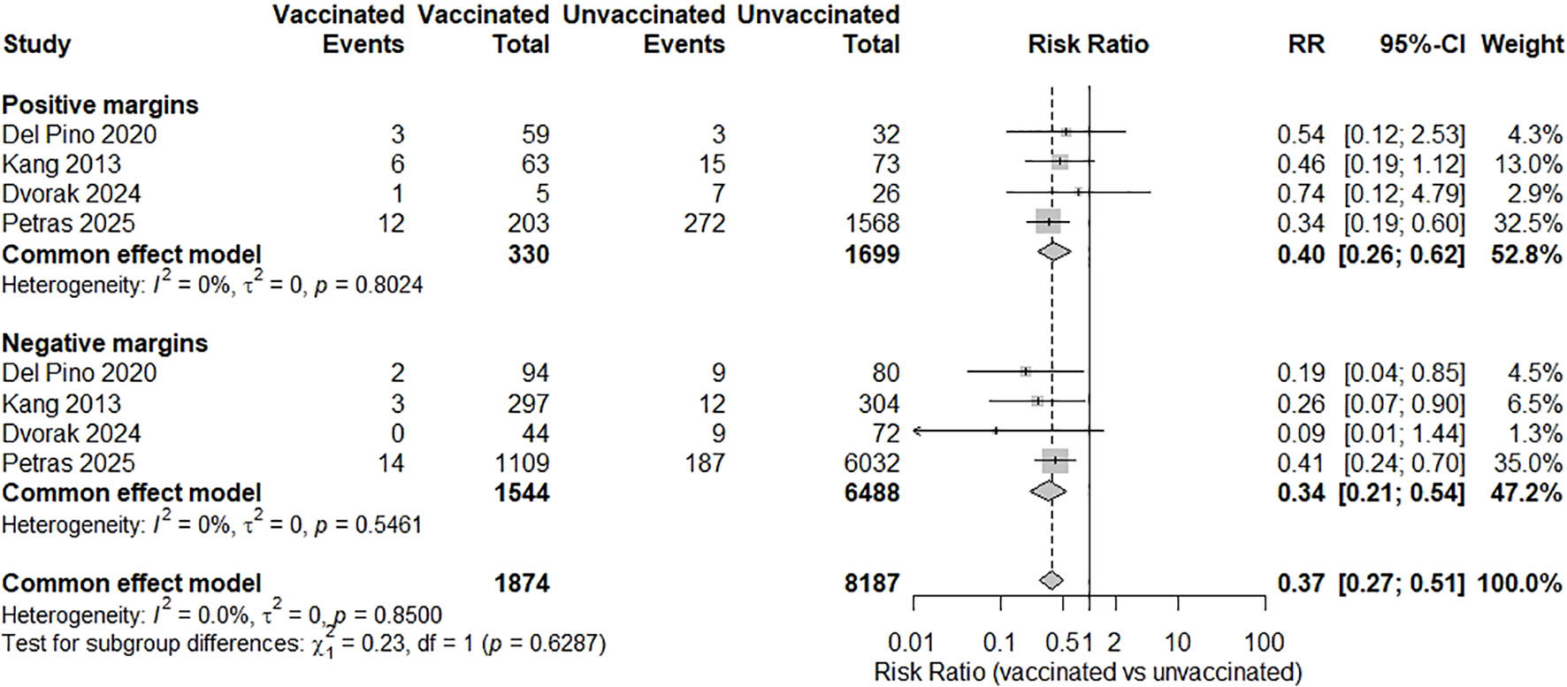
Subgroup meta-analysis of CIN2+ recurrence risk according to cone margin status. HPV vaccination was associated with a reduced recurrence risk both in women with positive margins (pooled RR = 0.40; 95% CI 0.26-0.62; 4 studies) and in those with negative margins (pooled RR = 0.34; 95% CI 0.21-0.54; 4 studies). No heterogeneity was observed within subgroups (I² = 0%). The fixed-effect model across all margin categories gave an overall RR of 0.37 (95% CI 0.27-0.51), and the test for subgroup differences was not significant (χ² = 0.23, df = 1, p = 0.63) (21, 28, 35, 36).

**Figure 5:**
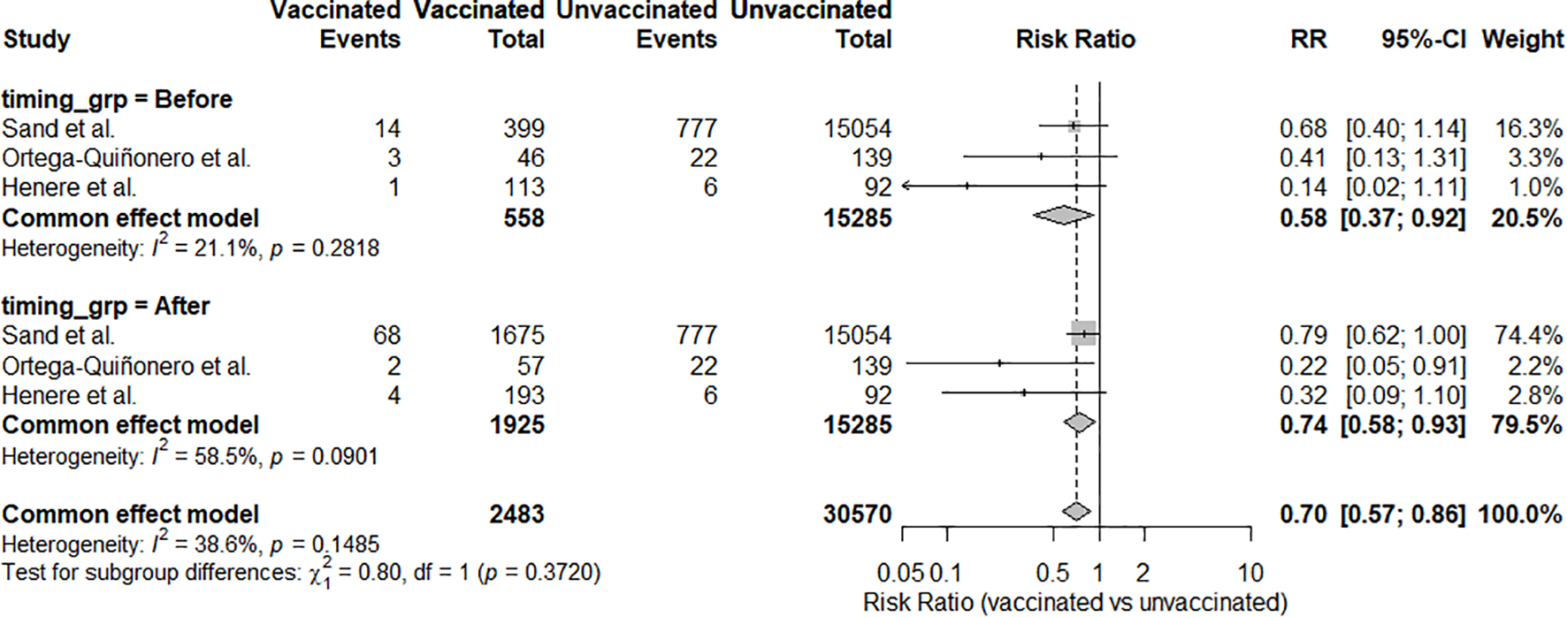
Forest plot comparing CIN2+ recurrence in vaccinated vs unvaccinated women, stratified by timing of vaccination (before vs after conization). Pooled RRs (common-effect, Mantel-Haenszel): before RR = 0.57 (95% CI 0.36-0.89; I² = 21.1%), after RR = 0.72 (95% CI 0.57-0.91; I² = 58.5%). No difference between subgroups (χ² = 0.89, p = 0.346) (25, 26, 33).

A meta-analysis of the 17 included studies demonstrated a statistically significant reduction in CIN2+ recurrence among those who received HPV vaccination following conization compared to unvaccinated controls (21–37). Using a random-effects model, the pooled RR for CIN2+ recurrence in vaccinated versus unvaccinated women was 0.38 (95% CI: 0.29-0.51; p < 0.0001), indicating a 62% relative risk reduction associated with vaccination. Moderate heterogeneity was observed across studies: I² = 58.6%, p for heterogeneity = 0.0012 (**Figure 2**). A subgroup meta-analysis evaluated whether vaccine valency modified the effect on CIN2+ recurrence. Two cohorts of the bivalent vaccine (n = 432 vaccinated; n = 445 controls) yielded a highly imprecise pooled RR of 0.50 (95% CI 0.06-4.08), with individual estimates of 0.18 and 1.55 (22, 26). Nine studies of the quadrivalent vaccine (n = 3,312 vs 16,441) produced a pooled RR of 0.37 (95% CI 0.23-0.58) (21, 23–27, 29, 30, 34). Three studies of the nonavalent vaccine (n = 757 vs 597) gave a pooled RR of 0.41 (95% CI 0.18-0.95) (33, 35, 37). The overall pooled effect across valency subgroups was RR = 0.39 (95% CI 0.28-0.56), with moderate heterogeneity (I² = 56.8%, τ² = 0.1866; p = 0.0045). The test for subgroup differences was not significant (χ² = 0.12, df = 2, p = 0.94) (**Figure 3**). A subgroup meta-analysis evaluated whether the effectiveness of HPV vaccination in preventing CIN2+ recurrence differed according to cone excision margin status. Four studies reported outcomes stratified by margin status, including 330 vaccinated and 1,699 unvaccinated women with positive margins and 1,544 vaccinated and 6,488 unvaccinated women with negative margins (21, 28, 35, 36). Among women with positive margins, the pooled RR for CIN2+ recurrence was 0.40 (95% CI 0.26-0.62; I² = 0%, p = 0.80). For women with negative margins, the pooled RR was 0.34 (95% CI 0.21-0.54; I² = 0%, p = 0.55). Overall, the fixed-effect model combining both subgroups yielded a pooled RR of 0.37 (95% CI 0.27-0.51; I² = 0%). The test for subgroup differences was not statistically significant (χ² = 0.23, df = 1, p = 0.63) (**Figure 4**). A subgroup meta-analysis of three studies compared vaccinated to unvaccinated women, stratified by timing of vaccination (25, 26, 33). Among 558 women vaccinated before conization, the pooled RR for CIN2+ recurrence was 0.57 (95% CI 0.36-0.89). Among 1,925 women vaccinated after conization, the pooled RR was 0.72 (95% CI 0.57-0.91). The test for subgroup differences was not significant (**Figure 5**). In the post-conization subgroup, most contributing cohorts defined time zero at surgery rather than at first vaccine dose; thus, these estimates may be partly affected by immortal-time bias and may slightly overstate the magnitude, though not the direction, of the protective effect of vaccination after conization.

### 3.6 Sensitivity analyses

Leave-one-out sensitivity analysis confirmed that no single study disproportionately influenced the overall pooled risk estimate. Excluding individual studies yielded pooled RRs ranging from 0.36 to 0.40, all of which remained statistically significant. The overall pooled effect remained stable at 0.38 (95% CI: 0.29-0.51). Notably, omission of Sand et al. reduced heterogeneity to 5%, indicating that this study was the main contributor to between-study variability (**Supplementary Figure 1**) (25). Visual inspection of the funnel plot suggested some asymmetry, with a relative paucity of small studies reporting null or harmful effects. Egger’s linear regression test confirmed significant funnel plot asymmetry (t = -3.42, df = 15, p = 0.0038), indicating the presence of small-study effects. Using the trim-and-fill method, seven potentially missing studies were imputed on the right side of the funnel (**Supplementary Figure 2**), and the adjusted pooled RR moved from 0.38 (95% CI 0.29-0.51) to 0.50 (95% CI 0.38-0.66), remaining statistically significant. This pattern suggests that small-study and publication bias may have modestly inflated the magnitude of the observed effect, with a more conservative but still clinically important reduction in recurrence risk. We therefore interpret 0.50 as a plausible lower-bound estimate of benefit and reflected this by grading the certainty of evidence as moderate, rather than high, in GRADE. Exploratory random-effects meta-regression provided additional insight into between-study variability. When study design (randomized trial, observational cohort, registry-based) was included as a moderator, heterogeneity decreased from I² ≈ 59% in the base model to a residual I² of ~40%, with an R² of 40.7% (i.e., design explained ~41% of the between-study variance). The test of moderators was statistically significant (QM = 6.18, df = 2, p = 0.0456). Compared with observational cohorts (estimated RR ≈ 0.29), randomized trials and registry-based studies showed attenuated, though still protective, effects (RR ≈ 0.55 and ≈ 0.53, respectively), consistent with smaller apparent benefits in more rigorous or population-based designs. By contrast, region did not explain heterogeneity (R² = 0%; QM = 2.14, p = 0.34), and timing of vaccination (pre vs. post vs. mixed) accounted for only ~1% of variance with a non-significant moderator test (R² = 1.2%; QM = 2.57, p = 0.28). Follow-up duration showed a borderline association (slope 0.0159 per month on the log RR scale; p = 0.076), corresponding to a modest attenuation of effect with longer follow-up, and explained ~33.5% of heterogeneity (R²), but confidence intervals were wide and these results should be interpreted cautiously (**Supplementary Table 1**).

### 3.7 Certainty of evidence (GRADE) assessment

Overall certainty for the primary outcome (CIN2+ recurrence) was judged Moderate, supported by a large, consistent effect that remained significant after trim-and-fill. Certainty for quadrivalent and nonavalent valency was Low, while bivalent was Very low (few studies/low events), and the margin-negative subgroup reached Moderate certainty (large, precise effect; I²=0%). By timing (vs unvaccinated), certainty was Moderate for the vaccinated before and Low for vaccinated after conization (observational base; low-moderate heterogeneity; no significant subgroup difference). Full judgments are provided in the Summary of Findings table (**Supplementary Table 2**).

### 3.8 Risk of bias assessment

RCTs were evaluated using the RoB 2 tool (15) while observational studies were assessed using the NOS (16). Among the RCTs, Hildesheim et al. was judged at low risk of bias across all domains (22). Karimi-Zarchi et al. was rated as having some concerns overall, due to limited reporting of the randomization process, deviations from intended interventions, and selective reporting (27). Pieralli et al. was considered at high risk of bias, mainly due to the non-blinded design and lack of adjustment for potential confounders (21). The phase 4 VACCIN trial by van de Laar et al. was judged as having some concerns overall: randomization, blinding, outcome measurement, and selective reporting were at low risk of bias, but there were some concerns related to missing outcome data for the primary endpoint (37). The thirteen observational studies achieved generally high NOS scores (range 6–9 out of 9), with all but one classified as high quality (7–9 points) and one study rated as moderate (6 points). These studies typically demonstrated clear selection of cohorts, histologically confirmed outcomes, and reasonable adjustment for confounders such as age, HPV persistence, and margin status. Large registry-based cohorts achieved the highest NOS scores, reflecting comprehensive data completeness and robust adjustment. Nevertheless, as with all non-randomized studies, residual confounding and selection bias (e.g., related to vaccine uptake) cannot be excluded. Across all included studies, outcome assessment was based on histologically confirmed CIN2+ recurrence, reducing the risk of detection bias. Discrepancies in risk of bias judgments were resolved by consensus. The detailed results of the risk of bias assessments are presented in **Supplementary Table 3** (RoB2 for RCTs) and **Supplementary Table 4** (NOS for observational studies).

## 4 Discussion

### 4.1 Principal findings

Across 17 studies (6665 vaccinated; 26,516 unvaccinated), HPV vaccination given around the time of conization was associated with a significant 50-70% reduction in CIN2+ recurrence (21–37). The pooled random-effects estimate was RR 0.38 with moderate heterogeneity, and the prediction interval suggested benefit in most future settings. Results were directionally consistent across designs and populations, with most individual studies favoring vaccination. By vaccine valency, all formulations showed benefit: the test for subgroup differences was not statistically significant (p = 0.94). Our valency analysis therefore supports that adjuvant HPV vaccination reduces post-conization CIN2+ recurrence across available vaccine types, with greatest precision for the quadrivalent formulation. We found no clear evidence that effectiveness differed by timing: in the three studies that reported both strategies, vaccination before and after conization each showed a reduction in recurrence, and the formal test for subgroup difference was not significant (p = 0.35), although confidence intervals, especially for pre-conization vaccination, were wide. Margin status did not statistically significantly modify benefit: vaccination was associated with a protective effect in both positive- and negative-margin cohorts, and the test for interaction between subgroups was non-significant (p = 0.63). Taken together, the evidence supports a clinically meaningful reduction in post-conization CIN2+ recurrence with adjuvant HPV vaccination, consistent with a prophylactic mechanism (prevention of new/re-infection) rather than clearance of established disease. Using GRADE, we judged the overall certainty for the reduction in CIN2+ recurrence to be Moderate, reflecting a large, consistent effect that persisted in sensitivity analyses. These findings support a clinical recommendation to offer adjuvant HPV vaccination after conization, while additional research will refine optimal implementation.

### 4.2 Comparison with existing literature

Our findings are concordant with the growing body of evidence over the past several years. High-quality studies and reviews have similarly reported that adjuvant HPV vaccination can substantially reduce the risk of cervical dysplasia recurrence. For example, a 2021 meta-analysis of 11 studies found that adding HPV vaccination to surgery was associated with roughly a 65% reduction in the risk of recurrent CIN2+ lesions (9). Another systematic review of 22 studies likewise concluded that adjuvant vaccination significantly lowers recurrence, particularly for lesions caused by HPV16/18: the high-risk types targeted by all current vaccines (10). These analyses align closely with our pooled estimate of vaccine efficacy, reinforcing this protective effect’s evidence. Our findings also align and extend the most recent quantitative syntheses. The 2023 PAVIVE meta-analysis of 20 studies reported an overall VE against recurrent CIN2+ of 69.5%, with the highest VE (78%) when vaccination was initiated on/after the day of conization; meta-regression found no effect of vaccine valency and suggested timing (post-excision) as the key modifier (11). After including in our study the most recent studies and a larger pooled sample (over 30,000 women), we were able to perform a stratified meta-analysis by timing and again found no significant difference between vaccinating before vs after conization, suggesting that any apparent timing effects in prior work likely reflect analytic/aggregation choices rather than true biological differences. Finally, our systematic review and meta-analysis is the first to assess the impact of surgical margin status in a pooled quantitative framework, showing clear benefit in women with negative margins and a similarly protective effect in those with positive margins, albeit with lower certainty. In conclusion, our analysis, which incorporates a broader range of studies, including more recent data, provides a more definitive assessment and suggests that the overall effect of adjuvant vaccination is indeed significant. While previous works have solely examined the impact of HPV vaccination after conization, our work provides the most comprehensive and up-to-date synthesis, adding subgroup analyses to include vaccine type, timing of vaccination, and surgical margin status. Compared to earlier analyses that lacked granularity or included older datasets, our review delivers the most up-to-date and clinically actionable insights for gynecologic oncologists, particularly regarding the applicability of vaccination across different surgical contexts.

### 4.3 Clinical implications

The demonstrated efficacy of HPV vaccination in preventing recurrences of high-grade CIN has important clinical implications. Integrating prophylactic HPV vaccination into the management of patients undergoing conization for CIN2/3 could meaningfully improve patient outcomes. In practice, this means that women (especially those who were never vaccinated or not fully vaccinated in youth) should be offered the HPV vaccine around the time of their excisional treatment for CIN. Doing so could reduce their absolute risk of recurrence from the typical ~10% (for untreated, unvaccinated cases) down to only ~3-4% or even lower, as evidenced by the studies (21–37). For clinicians, this translates into fewer patients returning with recurrent dysplasia on follow-up and, consequently, fewer repeat procedures such as second cone biopsies or hysterectomies. Reducing repeat excisional treatments is particularly valuable, as each additional cervical surgery increases risks such as cervical insufficiency and preterm birth in future pregnancies (33, 38–41). Thus, preventing recurrence not only spares patients the psychological stress of managing a repeat high-grade lesion but also preserves reproductive health. Adjuvant HPV vaccination is a simple but powerful intervention that could ultimately decrease progression to invasive cancer by eradicating recurrent precursor lesions. Importantly, the vaccine’s safety profile is well-established, and the benefits demonstrated in our meta-analysis far outweigh any minimal risks, making the risk-benefit profile highly favorable. Given this evidence, some professional guidelines have begun to incorporate adjuvant HPV vaccination into standard care (42). The American College of Obstetricians and Gynecologists (ACOG) now recommends discussing HPV vaccination for patients aged 27–45 undergoing treatment for CIN2+ who were not previously vaccinated, aligning with cervical pathology management guidelines (43). This reflects a growing consensus that offering the HPV vaccine in the post-conization setting is a prudent, evidence-based practice. Additionally, patients can be counseled that by getting vaccinated, they are significantly increasing the odds in favor of not developing another high-grade lesion. On a public health and economic level, widespread implementation of adjuvant HPV vaccination could yield cost savings and quality-of-life improvements by averting recurrences and the procedures and anxiety associated with them (44).

### 4.6 Research implications

While our analysis solidifies the evidence of benefit, it also highlights areas for further research. One key question is the optimal timing of vaccination relative to surgery. Our subgroup analysis did not find a significant difference between pre- versus post-conization vaccination, but this area warrants more investigation. Some preliminary data suggest that administering the first vaccine dose before or at the time of conization might confer the greatest protection (33). This finding implies that even a brief pre-treatment immunization interval could potentially help the immune system clear any residual HPV or prevent immediate reinfection. However, as of now, the optimal timing remains uncertain, and our results suggest that similar risk reductions are achieved when the vaccine is given before or shortly after (<12 months) conization. Ongoing and future clinical trials are expected to provide more definitive guidance on this issue. Another area for research is the effectiveness of adjuvant vaccination in specific subpopulations. For instance, immunocompromised patients (e.g., HIV positive patients or those on immunosuppressive therapy) have a higher baseline risk of recurrence and may respond differently to vaccination (45). Similarly, questions remain about the benefit of vaccination for those who were previously vaccinated but still developed CIN2 +. It is rare but possible for a vaccinated individual to present with CIN2/3, due to non-vaccine HPV types or incomplete vaccination: exploring whether an additional vaccine dose or a different vaccine (e.g., the nonavalent vaccine covering more types) can further reduce recurrence in such cases would be an interesting research direction. Furthermore, research could examine the broader impact of adjuvant HPV vaccination beyond cervical disease. Since HPV can cause vaginal, vulvar, and anal cancer, it is plausible that vaccinating a patient after treatment of a cervical lesion might also lower the risk of new HPV-related lesions in these other sites (46). Long-term follow-up studies that track any high-grade anogenital lesion or HPV-related malignancy as an outcome would help clarify if the protective effect extends to preventing multifocal disease. Additionally, studies with longer follow-up are needed to determine if adjuvant vaccination ultimately translates into a lower incidence of cervical cancer, and not just of pre-cancerous lesions. Finally, how prophylactic vaccination reduces recurrence remains to be clarified. The prevailing hypothesis is that vaccination prevents new HPV infections (including re-infection by the same strain after it was cleared by treatment), thereby reducing the chance of new lesions forming (47). In our meta-analysis, HPV vaccination was associated with a similar relative reduction in CIN2+ recurrence among women with both negative and positive surgical margins. This suggests that in margin-negative women, most subsequent lesions are likely driven by new or reactivated infections, whereas in margin-positive women vaccination cannot eradicate residual dysplastic foci but may still reduce the risk of additional vaccine-type infections and clinically overt recurrence. Recent large cohort data and the expanded margin-stratified evidence in our review further support a substantial benefit even in women with positive margins, although their absolute recurrence risk remains higher than that of margin-negative women despite vaccination. This points to a possible role for therapeutic HPV vaccines or immunotherapies in the future to complement prophylactic vaccines by actively eliminating established HPV-infected cells. Combining a prophylactic vaccine (to prevent new lesions) with a therapeutic approach (to clear residual disease) could help drive recurrence rates even lower in high-risk patients.

### 4.5 Limitations

Several limitations of our analysis should be addressed. First, most included studies were observational, so residual confounding and selection (indication) bias cannot be excluded despite generally high NOS scores and multivariable adjustments. In particular, vaccinated women may differ from controls in care-seeking or risk profiles; some cohorts also risk time-related/immortal-time bias when vaccination occurred after conization but follow-up accrued from surgery rather than vaccine date. Because we did not have access to individual time-to-event data, we were unable to reconstruct time-varying exposure models or to censor person-time before vaccination; instead, we explicitly classified these studies as at higher risk of time-related bias, downgraded the certainty of evidence for the “vaccinated after conization” subgroup in GRADE, and interpret those estimates as likely representing an upper bound of the true effect size. Importantly, the overall direction and statistical significance of the pooled effect were preserved in leave-one-out and registry-exclusion sensitivity analyses, suggesting that our main conclusion (reduction in recurrence risk) is robust despite this limitation. Outcome definitions varied (strict histologic CIN2+ recurrence vs composite “persistent/recurrent HSIL/HPV”), and surveillance schedules differed across studies, which may introduce detection bias and contribute to between-study variability. We observed moderate statistical heterogeneity in the main meta-analysis. Although predefined subgroup and sensitivity analyses supported robustness of the pooled effect, some heterogeneity is unavoidable given differences in design, populations, vaccine policies, and timing. Several subgroup estimates were based on few studies and low event counts, limiting precision and making the significant test for differences by valency hypothesis-generating rather than definitive. In addition, the presence of funnel-plot asymmetry and the attenuation of the pooled RR from 0.38 to 0.50 after trim-and-fill adjustment indicate that small-study/publication bias likely overestimates the magnitude of benefit, but not the direction; consequently, we have adopted a conservative interpretation of effect size and rated the overall certainty as moderate. Moreover, our review was restricted to full-text articles available in English, thus we cannot exclude language bias, and relevant data published in other languages may have been missed. Finally, safety reporting was sparse and non-standardized in observational series, limiting firm conclusions on rare adverse events (though no new safety signals emerged): our review cannot reliably quantify the risk of uncommon or long-term adverse events associated with adjuvant HPV vaccination. Future work should prioritize IPD meta-analysis or randomized trials with standardized outcomes, clear risk windows, genotype-specific endpoints, dose/timing adherence, and stratification by margin status and immune profile.

## 5 Conclusions

In this systematic review and meta-analysis of 17 randomized and observational studies, adjuvant HPV vaccination was consistently associated with a substantial reduction in CIN2+ recurrence after conization. Benefit was observed across vaccine formulations, when vaccination was given either before or shortly after surgery (although the evidence for pre-conization vaccination is based on relatively few events) and in women with both negative and positive cone margins. These findings support offering HPV vaccination as part of routine post-excisional management for eligible women as a pragmatic, low-burden intervention that may reduce repeat procedures, help preserve reproductive potential, and contribute to lowering the risk of invasive cervical cancer. Although most of the evidence is observational and some uncertainty remains regarding the optimal timing, vaccine valency, and specific high-risk subgroups, the available safety data are reassuring and the overall balance of benefits and harms is strongly favorable. Further individual-patient-data and prospective studies should refine implementation questions, but the current evidence already supports clear counseling that adding an HPV vaccine series around treatment is a practical step to decrease the likelihood of future high-grade cervical disease.

## Data Availability

The original contributions presented in the study are included in the article/**Supplementary Material**. Further inquiries can be directed to the corresponding author.
