# Supplementary material for: Impact of HPV vaccine on CIN2+ recurrence after conization: a systematic review and meta-analysis of vaccination timing, valency and surgical margins
Source: Front Oncol. 2026 Feb 25;16:1748972. doi: 10.3389/fonc.2026.1748972 (PMC12975468; doi:10.3389/fonc.2026.1748972)
Supplement: Supplementary file 1 [file DataSheet1.docx]

***Supplementary Material***

# Supplementary Files

**Supplementary File 1. Electronic Search Strategies.**

We systematically searched PubMed, Embase, Scopus and the Cochrane Library from 1 January 2013 to 30 November 2025. Searches were limited to human studies and English language. No study-design or language filters were applied at database level; restrictions to randomized trials and cohort studies were applied during screening.

**PubMed (searched 30 November 2025)**
Search string:

("Papillomavirus Vaccines"[Mesh]
OR "papillomavirus vaccines"[tiab]
OR "papillomavirus vaccine"[tiab]
OR "HPV vaccine"[tiab]
OR "HPV vaccines"[tiab]
OR "HPV vaccination"[tiab]
OR "human papillomavirus vaccin*"[tiab]
OR Gardasil[tiab]
OR "Gardasil 9"[tiab]
OR Cervarix[tiab])
AND
("Cervical Intraepithelial Neoplasia"[Mesh]
OR "cervical intraepithelial neoplasia"[tiab]
OR CIN2[tiab] OR CIN3[tiab]
OR "CIN 2"[tiab] OR "CIN 3"[tiab]
OR HSIL[tiab]
OR "high-grade squamous intraepithelial lesion"[tiab])
AND
(conization[tiab]
OR conisation[tiab]
OR "cervical conization"[tiab]
OR "cold knife conization"[tiab]
OR "loop electrosurgical excision"[tiab]
OR "loop electrosurgical excision procedure"[tiab]
OR LEEP[tiab]
OR "large loop excision"[tiab])
AND
(recurren*[tiab]
OR "treatment outcome"[Mesh])

Limits applied in PubMed: Publication date 2013/01/01–2025/11/30; Humans.

**Embase (Elsevier; searched 30 November 2025)**
Search string:

('papillomavirus vaccine'/exp
OR 'human papillomavirus vaccin*':ti,ab
OR 'hpv vaccin*':ti,ab
OR 'hpv vaccine*':ti,ab
OR gardasil:ti,ab
OR 'gardasil 9':ti,ab
OR cervarix:ti,ab)
AND
('cervix intraepithelial neoplasia'/exp
OR 'cervical intraepithelial neoplasia':ti,ab
OR cin2:ti,ab OR cin3:ti,ab
OR 'cin 2':ti,ab OR 'cin 3':ti,ab
OR hsil:ti,ab
OR 'high grade squamous intraepithelial lesion':ti,ab)
AND
('conization'/exp
OR conization:ti,ab
OR conisation:ti,ab
OR 'cervical conization':ti,ab
OR 'loop electrosurgical excision'/exp
OR 'loop electrosurgical excision':ti,ab
OR 'loop electrosurgical excision procedure':ti,ab
OR leep:ti,ab
OR 'large loop excision':ti,ab)
AND
(recurren*:ti,ab
OR 'treatment outcome'/exp)

Limits applied in Embase: Years 2013–2025; Humans.

**Scopus (Elsevier; searched 30 November 2025)**
Search string:

(TITLE-ABS-KEY("hpv vaccin*"
OR "hpv vaccine*"
OR "human papillomavirus vaccin*"
OR "papillomavirus vaccin*"
OR gardasil
OR "gardasil 9"
OR cervarix)
AND
TITLE-ABS-KEY("cervical intraepithelial neoplasia"
OR cin2
OR cin3
OR "cin 2"
OR "cin 3"
OR hsil
OR "high-grade squamous intraepithelial lesion")
AND
TITLE-ABS-KEY(conization
OR conisation
OR "cervical conization"
OR "cold knife conization"
OR leep
OR "loop electrosurgical excision"
OR "loop electrosurgical excision procedure"
OR "large loop excision")
AND
TITLE-ABS-KEY(recurren*))

Limits applied in Scopus: Publication year 2013–2025; document type Article/Article in press.

**Cochrane Library (CENTRAL and CDSR; searched 30 November 2025)**
Search string (search fields: title, abstract, keyword):

("hpv vaccin*"
OR "hpv vaccine*"
OR "human papillomavirus vaccin*"
OR "papillomavirus vaccin*"
OR gardasil
OR "gardasil 9"
OR cervarix)
AND
("cervical intraepithelial neoplasia"
OR cin2
OR cin3
OR hsil
OR "high-grade squamous intraepithelial lesion")
AND
(conization
OR conisation
OR "cervical conization"
OR leep
OR "loop electrosurgical excision"
OR "cold knife conization"
OR "large loop excision")
AND
(recurren*)

Limits applied in Cochrane: Year 2013–2025.

# Supplementary Tables

**Supplementary Table 1. Study-level covariates used in exploratory meta-regression.**

| **Covariates Table** | | | | | | |
| --- | --- | --- | --- | --- | --- | --- |
| **Study** | **Design** | **Region** | **Timing** | **Followup_months** | **n_vac** | **n_ctrl** |
| Kang 2013 | Cohort | Asia | post | 24.0 | 360 | 377 |
| Hildesheim 2016 | RCT | LatinAmerica | pre | 56.7 | 362 | 375 |
| Ghelardi 2018 | Cohort | Europe | post | 36.0 | 172 | 172 |
| Pieralli 2018 | RCT | Europe | post | 36.0 | 89 |  |
| Sand 2020 | Registry | Europe | mixed | 54.0 | 2074 | 89 |
| Ortega 2019 | Cohort | Europe | mixed | 24.0 | 103 | 15054 |
| Karimi-Zarchi 2020 | RCT | Asia | post | 24.0 | 138 | 139 |
| Del Pino 2020 | Cohort | Europe | mixed | 22.4 | 153 | 104 |
| Bogani 2020 | Cohort | Europe | post | 60.0 | 100 | 112 |
| Petrillo 2020 | Cohort | Europe | post | 6.0 | 182 | 200 |
| Gomez 2021 | Cohort | Europe | post | 27.0 | 160 | 103 |
| Casajuana 2022 | Cohort | Europe | post | 33.0 | 277 | 171 |
| Henere 2022 | Cohort | Europe | mixed | 20.0 | 306 | 286 |
| Chen 2023 | Cohort | Asia | post | 24.0 | 148 | 92 |
| Dvorak 2024 | Cohort | Europe | post | 30.0 | 49 | 273 |
| Petras 2025 | Registry | Europe | mixed | 50.4 | 1590 | 8464 |
| van de Laar 2025 | RCT | Europe | post | 24.0 | 402 | 407 |

RCT: randomized controlled trial

**Supplementary Table 2. Summary of Findings (GRADE):** HPV vaccination after conization.

| **Outcome** | **Participants (studies)** | **Relative effect (RR, 95% CI)** | **Absolute risk without vaccine (per 1,000)** | **Absolute risk with vaccine (per 1,000) [95% CI] → ARR** | **Certainty (GRADE)** | **Reasons / notes** |
| --- | --- | --- | --- | --- | --- | --- |
| CIN2+ recurrence (overall) | 33,181 (17) | 0.38 (0.29–0.51) | 94 | 35.7 (27.3–47.9) → 58.3 fewer/1,000 (NNT≈17) | Moderate ⊕⊕⊕⊝ | Start low (observational), upgrade for large effect; trim-and-fill still significant. (a,e; see b) |
| CIN2+ recurrence (trim-and-fill adjusted) | 22,318 (+7 imputed) | 0.50 (0.38–0.66) | 94 | 47.0 (35.5–61.9) → 47 fewer/1,000 (NNT≈21) | Moderate ⊕⊕⊕⊝ | Addresses small-study effects; effect persists with acceptable precision. (a,b) |
| Valency – Quadrivalent (4v) | 19,753 (9) | 0.37 (0.23–0.58) | 94 | 34.8 (21.6–54.5) → 59.2 fewer/1,000 (NNT≈17) | Low ⊕⊕⊝⊝ | Observational base; heterogeneity/publication bias suspected. (a,b,d) |
| Valency – Nonavalent (9v) | 1354 (3) | 0.41 (0.18–0.95) | 94 | 38.5 (16.9–89.3) → 55.5 fewer/1,000 | Low ⊕⊕⊝⊝ | Few studies/low events. (a,d) |
| Valency – Bivalent (2v) | 877 (2) | 0.50 (0.06–4.08) | 94 | 47.0 (5.6–383.5) → effect extremely uncertain | Very low ⊕⊝⊝⊝ | Imprecision/indirectness. (a,d,f) |
| Margin-negative | 8032 (4) | 0.34 (0.21–0.54) | 94 | 32.0 (19.7–50.8) → 62 fewer/1,000 (NNT≈16) | Moderate ⊕⊕⊕⊝ | Large, precise effect; I²=0%; upgrade for large effect. (e) |
| Margin-positive | 2029 (4) | 0.40 (0.26–0.62) | 94 | 37.6 (24.4–58.3) → 56.4 fewer/1,000 | Low ⊕⊕⊝⊝ | Small n and imprecision (CI includes no effect). (a,d) |
| Timing – Vaccinated *before* conization | 558 (3) | 0.57 (0.36–0.89) | 94 | 53.6 (33.8–83.7) → 40.4 fewer/1,000 (NNT≈25) | Moderate ⊕⊕⊕⊝ | Observational base; I² ≈ 21% (low). (a) |
| Timing – Vaccinated *after* conization | 1,925 (3) | 0.72 (0.57–0.91) | 94 | 67.7 (53.6–85.5) → 26.3 fewer/1,000 (NNT≈38) | Low ⊕⊕⊝⊝ | Observational base; moderate heterogeneity (I² ≈ 59%). (a,d) |

Relative effects are risk ratios (RR) with 95% CIs. Absolute risks assume a baseline recurrence risk of 94 per 1,000 unvaccinated women. Certainty was rated with GRADE considering risk of bias, inconsistency, indirectness, imprecision, and publication bias. Footnotes: a Predominantly observational evidence (non-randomized cohorts and registry-based analyses). b Small-study/publication bias: funnel-plot asymmetry and trim-and-fill adjustment attenuated the pooled RR from 0.38 to 0.50, but the effect remained statistically and clinically significant; certainty was therefore downgraded for publication bias, while maintaining an overall moderate rating. d Heterogeneity and/or imprecision in subgroup estimates. e Large effect (RR ≤ 0.5) and consistent direction of benefit across studies → one-level upgrade. f Indirectness for valency (sparse data, no head-to-head comparisons between vaccine types).

**Supplementary Table 3.** Risk of bias assessment of randomized controlled trials (RCTs) evaluating HPV vaccination after conization, using the RoB2 tool. Traffic light colors indicate overall judgment for each domain: 🟩 green = low risk, 🟧 orange = some concerns, 🟥 red = high risk.

| **Study** | **Randomization** | **Deviations from intended interventions** | **Missing outcome data** | **Measurement of outcome** | **Selection of reported results** | **Overall** |
| --- | --- | --- | --- | --- | --- | --- |
| **Karimi-Zarchi** | 🟧 | 🟧 | 🟩 | 🟩 | 🟧 | 🟧 |
| **Pieralli** | 🟧 | 🟥 | 🟩 | 🟩 | 🟧 | 🟥 |
| **Hildesheim** | 🟩 | 🟩 | 🟩 | 🟩 | 🟩 | 🟩 |
| **Van de Laar** | 🟩 | 🟩 | 🟧 | 🟩 | 🟩 | 🟧 |

**Supplementary Table 4.** Newcastle–Ottawa Scale (NOS) assessment of observational studies evaluating HPV vaccination after conization. Each study was scored across three domains: Selection (maximum 4 points), Comparability (maximum 2 points), and Outcome (maximum 3 points). Blue stars indicate awarded points within each domain. The total NOS score ranges from 0 to 9, with higher scores indicating better methodological quality. All but one study were rated as high quality (7–9 points), while one was rated as moderate quality (5–6 points).

| **Study** | **Selection**  **(max 4)** | **Comparability**  **(max 2)** | **Outcome**  **(max 3)** | **Total**  **(0–9)** |
| --- | --- | --- | --- | --- |
| **Kang et al.** (2013)  Retrospective | **★★★★** | **★**☆ | **★★★** | **8** |
| **Ghelardi et al. (SPERANZA)** (2018)  Prospective | **★★★★** | ☆☆ | **★★**☆ | **6** |
| **Gómez de la Rosa et al.** (2021)  Longitudinal observational | **★★★★** | **★**☆ | **★★★** | **8** |
| **Bogani et al.** (2020)  Retrospective | **★★★★** | **★★** | **★★★** | **9** |
| **Casajuana-Pérez et al. (VENUS)**  (2022)  Retrospective | **★★★★** | **★**☆ | **★★★** | **8** |
| **Ortega-Quinonero et al.**  (2019)  Retrospective | **★★★★** | **★**☆ | **★★★** | **8** |
| **Del Pino et al.** (2020)  Prospective | **★★★★** | **★**☆ | **★★★** | **8** |
| **Henere et al.** (2022)  Observational | **★★★★** | ☆☆ | **★★★** | **7** |
| **Chen et al.** (2023)  Prospective | **★★★★** | **★**☆ | **★★★** | **8** |
| **Dvořák et al.** (2024)  Retrospective | **★★★★** | **★★** | **★★★** | **9** |
| **Petrillo et al.**  (2020)  **Retrospective** | **★★★★** | **★☆** | **★★★** | **8** |
| **Sand et al.** (2020)  **Nationwide population-based** | **★★★★** | **★★** | **★★★** | **9** |
| **Petras et al.** (2025)  **Retrospective** | **★★★★** | **★★** | **★★★** | **9** |

# Supplementary Figures


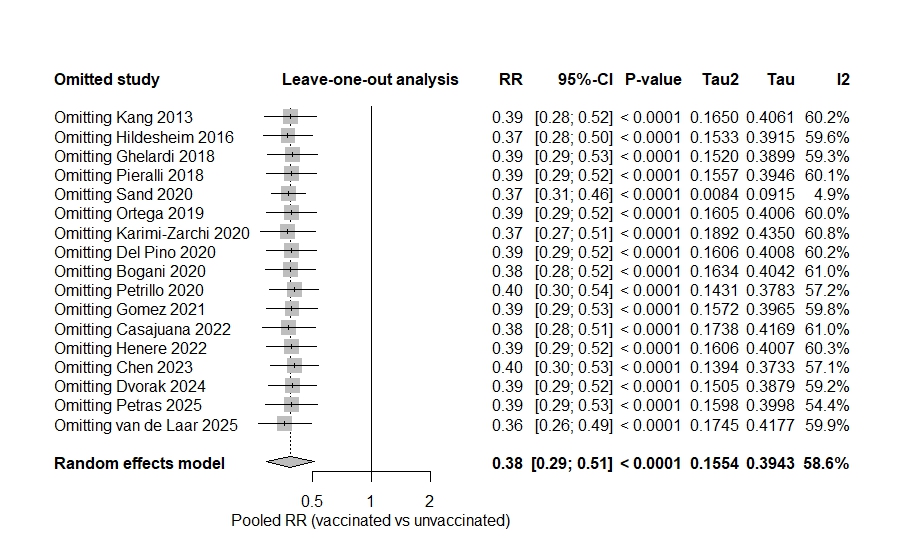


**Supplementary Figure 1.** Leave-one-out sensitivity analysis of the pooled risk ratio for CIN2+ recurrence after HPV vaccination. Exclusion of individual studies had minimal impact on the overall effect estimate (range of pooled RR: 0.29–0.51) (21-37). Omitting the large registry-based study by Sand et al. reduced heterogeneity to 4.9% (I²), suggesting it was the primary source of between-study variability (25).

**
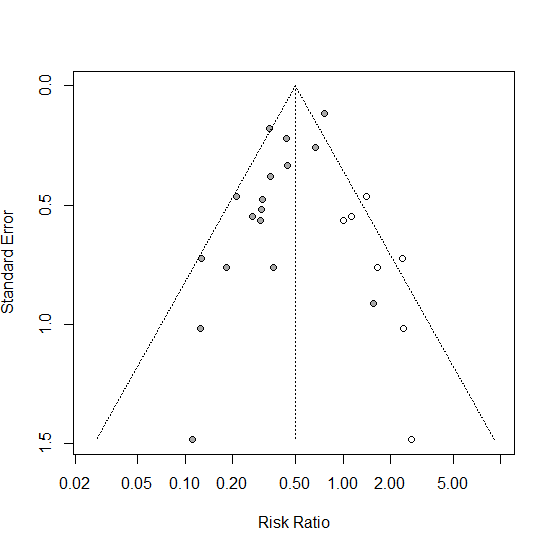
**

**Supplementary Figure 2**. Funnel plot assessing publication bias in the meta-analysis of HPV vaccination and CIN2+ recurrence. Egger’s linear regression test confirmed significant funnel plot asymmetry (t = –3.42, df = 15, p = 0.0038), indicating the presence of small-study effects. Trim-and-fill analysis imputed seven potentially missing studies. The adjusted pooled RR moved from 0.38 (95% CI 0.29–0.51) to 0.50 (95% CI 0.38–0.66), remaining statistically significant.
